# The role of γ-aminobutyric acid and salicylic acid in heat stress tolerance under salinity conditions in *Origanum vulgare* L.

**DOI:** 10.1371/journal.pone.0288169

**Published:** 2023-07-07

**Authors:** Meisam Keshtkar Garoosi, Forough Sanjarian, Mehrdad Chaichi

**Affiliations:** 1 Plant Bioproducts Department, Institute of Agricultural Biotechnology (IAB), National Institute of Genetic Engineering and Biotechnology,Tehran, Iran; 2 Department of Seed and Plant Improvement Research, Hamedan Agricultural and Natural Resources, Research and Education Center, Agricultural Research, Education and Extension Organization, Hamedan, Iran; Bangabandhu Sheikh Mujibur Rahman Agricultural University, BANGLADESH

## Abstract

*Origanum vulgare* L., a medicinal and aromatic herb, has been used for hundreds of years. This plant contains valuable chemical compounds that can be used as medicine for treatment. On the other hand, a gradual increase in the planet’s average temperature could negatively affect the growth and the composition of the *O*. *vulgare*. For this reason, in this study, the effect of two protective compounds, namely salicylic acid (SA) and gamma-aminobutyric acid (GABA), on temperature and salinity stress conditions was investigated. Oregano plants were grown at 23/12°C temperature as control and 27/16°C heat stress in the greenhouse (16/8 h photoperiod) for one months. The plants were treated with GABA and SA and subjected to salt stress for 30 days. Subsequently, the plant’s physiological, biochemical, and phytochemical characteristics were examined. The results showed that all studied traits (in control and treated samples) were significantly different at 27°C, from 23°C. In addition, the highest amount of thymol and carvacrol were detected from plants grown at 27°C. In regards to salinity, stressed- plants had less damage to membrane stability and H_2_O_2_ level, when treated with GABA or SA. This study revealed that both SA and GABA compounds had an exellent protective effect on temperature and salt stress on *O*. *vulgare*. Based on enzyme-pigment evaluations and secondary metabolites, SA showed a better protective effect on temperature effects and GABA in a saline environment. In general, using these compounds can provide better conditions for the growth and preservation of *O*. *vulgare* chemical compounds. However, it certainly requires more experiments to find the signal pathways involved in these processes.

## Introduction

*Origanum vulgare* L. is a fragrant perennial herb in the Lamiaceae family that is rich in essential oil. It is found in the Mediterranean and western Eurasia, where it is endemic. *O*. *vulgare* is an herb that is widely used as a flavoring agent all over the world, particularly in Asian cuisine. Flavonoids, flavonols, tannins, cholesterol, and phenolic glycosides are a few of the medicinally beneficial components found in the aerial part of this plant [1,2].

Herbs have long been utilized to promote good health. Because of their importance to human health, medicinal plants are an important topic in medical research. To use medicinal plants, one must first be familiar with their traditional and therapeutic uses. *O*. *vulgare* has anti-tumor, anti-diarrheal, and anti-inflammatory effects. Additionally, the herb is an analgesic, anti-cough, anti-parasitic, expectorant, diuretic, and blood-pressure-lowering agent. *O*. *vulgare* extract has antibacterial, antioxidant, and hepatoprotective properties and also contains a high polyphenol content [3].

Climate change will affect the structure and function of ecosystems, favoring species that can resist higher temperatures and more arid conditions [4]. One of the destructive effects of climate change is accelerating the development of soil salinity. This adversity and the areas affected will extend in the near future [5]. Salinity exerts its detrimental effect on plants by disturbing the morphological, physiological, biochemical, and molecular processes [6]. Plants have developed numerous adaptive mechanisms to cope with environmental stress. They response to stress by accumulating protectant primary and secondary metabolites, altering their phytohormone signaling and enhancing own antioxidant defense systems [7,8]. Phytohormones are endogenous messenger molecules that regulate different defensive responses to several abiotic stresses, including heat and salinity [9,10]. Exogenous applications of plant hormones previous to or parallel with stress significantly recover stress-induced damage and enhance plant tolerance [11,12].

Salicylic acid (SA), a phenolic phytohormone, regulates plant growth and development. In addition, recent studies demonstrate that SA has a role in plant responses various of environmental stresses [13].

GABA, γ- aminobutyric acid, is a non-protein amino acid that helps plants survive drought, heavy metal pollution, and saline environments. GABA claims that it regulates the plant cell’s redox status and osmotic potential and mitigates abiotic stress. It can restrict stomatal opening and transpiration while also influencing the release of the tonoplast-localized anion transporter [14]. The Hasan et al. research team compiled a list of scholarly works on GABA-induced drought resistance in plants [15]. Plants’ metabolic and signaling capabilities have been shown to assist plants in surviving drought. Furthermore, some research studies revealed that exogenous GABA application during salt stress conditions reduces the plant oxidative damage. γ- aminobutyric acid boosts photosynthesis rate, increases the leaf water content and enhances scavenging free radicals via interaction with plant growth regulators [16–18].

It has been made clear that exogenous applications of plant signaling molecules previous or parallel with stress significantly recover stress-induce damage and enhance plant tolerance. Thus, this study aimed to explore the effects of SA and GABA on physiological, biochemical, and secondary metabolites in *O*. *vulgare* in terms of enhancing heat and osmotic stress tolerance.

## Materials and methods

### Plants and treatments

*Origanum vulgare* seeds and cuttings were purchased from Zarrin Giah Production Company (www.zarringiah.com). In preliminary research, NaCl (50, 75, 100, 150, 175 mM) and GABA (0.5, .0.7, 1.0, 1.5 and 2.0 mM) were separately applied to seedling via root drenching and foliar spray application. The results showed no fatal effects on seedlings by NaCl concentration up to 100 mM. Moreover application of 1 mM GABA exerted maximum benefits for seedling growth. The concentration of 1mM salicylic acid was selected from earlier trials [19]. The three month-old cutting plants were then planted in 5-liter pots filled with a 2:1 mixture of air-dried loam soil (pH 7) and sand. These plants were raised in greenhouse conditions (23°C and 16/8 h.d^-1^ photoperiod) for one month. Afterwards, half of the pots were maintained in a controlled environment at 23°C (control) while the other half were subjected to 27°C heat stress. As a form of treatment, GABA and salicylic acid were sprayed on the plant, and salinity treatment involved drenching the roots with the salt solution. The two temperature groups (23 and 27°C) received weekly treatments for one month. Finally, there were the following treatments for separate experiments: (a) 23°C, no salt stress, no SA or GABA, (b) 23°C, no salt stress, no SA + 1 mM GABA, (c) 23°C, no salt stress, no GABA + 1 mM SA, (d) 23°C, 100 mM NaCl, no SA or GABA, (e) 23°C, 100 mM NaCl, no SA + 1 mM GABA, (f) 23°C, 100 mM NaCl, no GABA + 1 mM SA, (g) 27°C, no salt stress, no SA or GABA, (h) 27°C, no salt stress, no SA + 1 mM GABA, (i) 27°C, no salt stress, no GABA + 1 mM SA, (j) 27°C, 100 mM NaCl, no SA or GABA, (k) 27°C, 100 mM NaCl, no SA + 1 mM GABA, (l) 27°C, 100 mM NaCl, no GABA + 1 mM SA

### Total fresh and dry weights

Fresh weight was measured accurately immediately after harvest. Dry weights were recorded after those samples were oven-dried at 70°C for 72 h or until weights were constant.

### Photosynthetic pigments determination

Powdered leaves material was extracted several times with cold absolute acetone until completely uncolored. The extracts were then centrifuged for 5 min at 10,000 × g. The contents of chlorophyll a (Chl a), chlorophyll b (Chl b), and carotenoids (Car) were measured spectrophotometrically (Specord 50, Analytik Jena, Germany) at 470, 661.6, and 644.8 nm and calculated according to [20]. The fluorescence meter (*Pocket PEA*, *ansatech*, Norfolk, UK) was used to figure out the photochemical efficiency (F0 and Fm) of a single leaf that had been in the dark for 30 min.

### Antioxidant enzymes activities assay

200 mg of liquid nitrogen freezed shoot samples were grind in 2 mL phosphate buffer solution (100 mM) and centeifuged at 4°C for 20 min at 10,000 × g. The supernatant was tested for total protein concentration and enzymes activities. The Bradford method was used to determine the total protein concentration using BSA as a standard reference. Peroxidase (POD) activity was measured by monitoring the H_2_O_2_-dependent oxidation of guaiacol at 470 nm. One unit of enzyme activity was defined as the amount of enzyme that caused the formation of 1 μM of tetraguaiacol per minute. Superoxide dismutase (SOD) activity was measured in term of the enzyme’s ability to inhibit of the photochemical reduction of nitroblue tetrazolium (NBT) in the presence of riboflavin. SOD activity was defined as the amount of sample required for 50% inhibition [21]. Polyphenol oxidase activity was was monitored by measuring the increase in absorbance for one minute using pyrogallol as substrate. The amount of purpurogallin formed on PPO activity were estimated by measuring the absorbance at 420 nm [22].

### Antioxidant components

A 0.5 mg of plant tissues were soacked in 4 mL of absolute methanol at at 37°C for 30 min. The homogenate was centrifuged for 5 min at 10,000 × g. The obtained supernatant was stored in 4°C until the experiments were performed. The total phenol concentration was mixed with a 10% Folin-Ciocalteu reagent (diluted 1:10) and 1 M sodium carbonate. The reaction mixture was left for 30 min at room temperature, and the absorbance was measured at 750 nm with UV/VIS spectrophotometer (Specord 50, Analytik Jena, Germany). The gallic acid standard curve was used to evaluate total phenol content in the samples [23]. Aluminum chloride colorimetric method was used for flavonoids determination using the Quercetin equivalent (μm) of the extract [24]. Anthocyanins were extracted using acidic methanol (methanol 99.5 percent and HCl 1 percent, ratio 99 at 1). Preserve the solvent, the mixture was kept at 4°C for 24 h, with no light exposure. The absorbance of the supernatant at 530 nm was measured after centrifugation at 4,000 × g for 10 min [25].

### Oxidative stress indicators

The H_2_O_2_ content of leaf samples was colorimetrically measured as described by [26]. Leaf samples were extracted with cold 0.1% TCA to determine the H_2_O_2_ levels and then centrifuged for 20 min at 12,000 × g. The 0.5 mL of supernatant was treated with a 10 mM potassium phosphate solution and 1 mL of KI. The solution’s absorbance was observed at a 390 nm. Lipid peroxidation of membranes were analyzed using [26] to measure the malondialdehyde (MDA) content. The tissues were extracted with distilled water in boiling water bath for 30 min for estimated prolin content. Then the acidic ninhydrin reagent was employed to initiate a reaction to form a chromophore. Calibration were made with 1 mM L-proline as a standard [25].

### Essential oils (EO) analysis

The aerial parts of the plants were dried at ambient temperature for 6 to 12 days at 25°C and then ground. Samples (30 mg) were submitted to hydro distillation using a Clevenger-type apparatus for three hours. The oils were dried with anhydrous sodium sulfate and stored at 4°C until they could be analyzed. Essential oil composition was characterized using GC–flame ionization detection (GC-FID) and GC–MS analyses. Gas chromatography (GC) analysis was performed using a Thermo-Trace GC system (Thermo Electron, San José, CA, USA) with a flame ionization detector (FID). The FID equipped with a split/spitless injector (250°C), a fused silica capillary DB-5 column (30 m length × 0.25 mm internal diameter; 0.25 μm film thickness) and a flame ionization detector (250°C). Nitrogen was used as the carrier gas at a flow rate of 1.1 mL/min; the oven temperature program was 60–250°C. The injector temperature was 250°C and 1 μL of oil sample was injected in the split mode with the split ratio of 10:1. The oil samples were analyzed on a Thermo-Trace GC–MS system (Thermo Electron, San José, CA, USA), equipped with a DB-5 silica column containing 5% phenyl–95% methyl polysiloxane and a mass spectrometer detector. The oven temperature program was 40–460°C; injector temperature: 250°C. Helium was used as the carrier gas at rate of 1.1 mL/min. Ionization voltage was 70 eV, and ion source was 200°C.

The mass spectra and retention indices of various constituents of essential oils were found and incorporated into the MS computer library based on published literature.

### Statistical analysis

Statistical analysis of data was performed using a factorial experiment in a randomized complete block design with three replications. The first factor included four levels of GABA (0 and 1 mM) and SA (0 and 1 mM) treatments in a total of four levels. The second factor, salinity, was applied at two levels (0 and 100 mM NaCl) through root drenching. The third factor was the temperature, which was applied by placing the plants in the growth chamber at temperatures of 23 and 27°C on two levels. Statistical analysis of data was performed using SAS software and GLM procedure. The examined traits included fresh and dry weights, chlorophyll (a, b), total carotenoids, antioxidant enzymes activities (POD, PPO, SOD), total phenolic and flavonoid contents, H_2_O_2_, MDA and essential oil composition. In order to simultaneously compare the evaluated traits with the applied treatments, principal component analysis was used. For this purpose, the fviz-pca function in the factoextra R package ver. 1.0.7 [27] was used to make it possible to view the biplot of treatments with variables.

## Results

The chemical constituents of *O*. *vulgar* essential oil was analyzed by GC-MS and identified of 27 compounds, which are listed in S1 Table (GC-MS chromatogram is represented in S1 Fig). S1 Table showed that the essential oil was mainly composed of: p-Cymene (30.80%), γ-Terpinene (17.83%), Thymol (5.42%), Carvacrol (4.56%), Thymol methyl ether (2.99%) and Carvacrol methyl ether (5.26%). The amount of these compounds were affected by all treatments (S2 Fig).

### Significant relationship between salinity and temperature with SA and GABA treatments

The present study aimed to explore the effects of three distinct treatments, namely GABA or SA, salinity, and temperature, as well as their interactions on various plant traits. Results from analysis the data variance revealed that 3-way interactions were statistically significant for nearly all examined characteristics, except for chlorophyll content and plant stem weight (Table 1). In other words, slight changes in temperature and salinity can cause varied reactions in the observed characteristics when treated with external treatments like SA or GABA. However, when viewed from a different standpoint, the 2-way interactions of exogenous treatment of SA or GABA with temperature or salinity showed no significant influence on the attributes related to shoot weight. Instead, they brought about notable shifts in the activity of enzymes and the amount of metabolites. In particular, the interaction between salt and temperature had no significant effect on photosynthetic traits or shoot weight. Consistently high levels of these features were observed at 27°C without any salt (Table 1).

**Table 1:** Analysis of variance of the effect of temperature, salt and foliar application of SA and GABA on studied traits in *O*. *vulgare*.

Traits	*P* values							
	Treatment	Salt	Temp	Treatment × Salt	Treatment × Temp	Salt × Temp	Treatment × Salt × Temp	Mean ± SE
Total protein	0.0044	0.0519	< 0.0001	< 0.0001	0.0010	< 0.0001	0.0025	1.69 ± 0.086
Peroxidase	< 0.0001	0.4299	< 0.0001	0.0003	0.0002	< 0.0001	< 0.0001	0.0028 ± 0.00027
PPO	< 0.0001	0.0005	< 0.0001	< 0.0001	< 0.0001	< 0.0001	< 0.0001	0.0034 ± 0.00018
SOD	< 0.0001	< 0.0001	< 0.0001	< 0.0001	< 0.0001	< 0.0001	< 0.0001	16.55 ± 0.879
H_2_O_2_	0.0008	0.0906	< 0.0001	0.0478	< 0.0001	0.0296	< 0.0001	66.056 ± 7.90
MDA	< 0.0001	< 0.0001	< 0.0001	< 0.0001	< 0.0001	< 0.0001	< 0.0001	0.0862 ± 0.0346
Phenol	< 0.0001	< 0.0001	< 0.0001	< 0.0001	< 0.0001	< 0.0001	< 0.0001	3.76 ± 0.0019
Flavonoids	< 0.0001	0.0005	0.0017	< 0.0001	< 0.0001	0.9785	< 0.0001	0.456 ± 0.013
Anthocyanins	< 0.0001	< 0.0001	0.0032	< 0.0001	0.1542	0.0573	0.0194	5.11 ± 0.224
Chl a	< 0.0001	< 0.0001	< 0.0001	< 0.0001	0.0003	0.5877	0.0025	24.81 ± 0.67
Chl b	0.0018	< 0.0001	0.0005	0.0334	< 0.0001	0.1282	0.0060	10.65 ± 0.244
Total Ch	< 0.0001	< 0.0001	< 0.0001	< 0.0001	0.0751	0.0897	< 0.0001	35.44 ± 0.817
Carotenoids	< 0.0001	< 0.0001	< 0.0001	< 0.0001	0.2013	0.0194	0.0031	2346.46 ± 58.08
F_m_	< 0.0001	0.0012	< 0.0001	0.0004	0.0026	0.0791	0.6852	221.83 ± 2.61
F_0_	0.1310	0.1213	0.2283	0.1862	0.0552	0.3035	0.7131	106.58 ± 2.71
Fresh weight	0.3766	0.9072	< 0.0001	0.7038	0.0289	0.0855	0.2398	376.22 ± 22.224
Dry weight	0.2815	0.5531	0.0015	0.6644	0.9842	0.6507	0.9408	101.669 ± 6.685
Biomass	0.0393	0.4108	0.8322	0.1402	0.1122	0.0190	0.1572	27.682 ± 1.502
p-Cymene	< 0.0001	< 0.0001	< 0.0001	< 0.0001	< 0.0001	< 0.0001	< 0.0001	26.40 ± 0.710
γ-Terpinene	< 0.0001	< 0.0001	< 0.0001	< 0.0001	< 0.0001	< 0.0001	< 0.0001	21.39 ± 0.626
Thymol, methyl ether	0.7378	< 0.0001	< 0.0001	< 0.0001	< 0.0001	< 0.0001	< 0.0001	3.682 ± 0.257
Carvacrol, methyl ether	< 0.0001	0.0003	< 0.0001	< 0.0001	< 0.0001	< 0.0001	< 0.0001	4.933 ± 0.198
Thymol	< 0.0001	< 0.0001	< 0.0001	< 0.0001	< 0.0001	< 0.0001	< 0.0001	5.208 ± 0.332
Carvacrol	< 0.0001	< 0.0001	0.0323	< 0.0001	< 0.0001	< 0.0001	< 0.0001	2.743 ± 0.235

**Fig 1:**
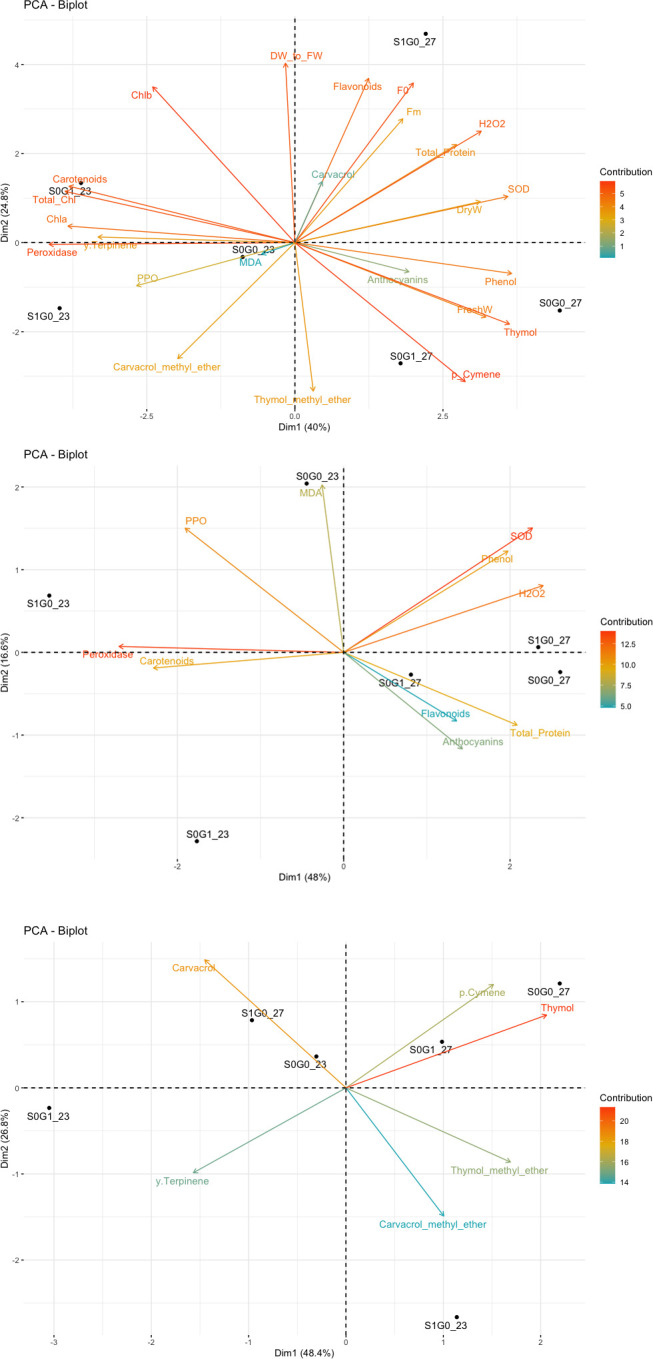
Bioplots at temperatures of 23 and 27°C. A: Biplot for the average of all measured traits at 23 and 27°C separately for treatments. B: Biplot for the average of traits related to enzymatic traits and pigments measured at 23 and 27°C separately for treatments. C: Biplot for the average traits related to the production of secondary metabolites at 23 and 27°C separately for treatments. S0: no SA, S1: 1 mM SA, G0: no GABA, G1: 1 mM GABA, 23: 23°C, 27: 27°C.

The biplot generated from the mean values of all traits measured at 25°C and 27°C for external SA and GABA treatments illustrates that there is a clear separation between the two temperatures in all external treatments (Fig 1A). To gain a better understanding of these results, the measured traits were categorized into two groups: enzyme-pigment (inclulding peroxidase, polyphenol oxidase and superoxidase activities; and the amount of hydrogen peroxide, malondialdehyde, total phenol, flavonoids, anthocyanin, carotenoid and protein) and secondary metabolites (such as p-Cymene, γ-Terpinene, Thymol, Thymol methyl-ether, Carvacrol and Carvacrol methyl ether).

### Interaction between enzymatic characteristics and pigments with SA and GABA treatments

The findings indicate that when the SA treatment was applied at 27°C, there was a rise in the activity of superoxide dismutase (11%) and the levels of total phenol (44%) and hydrogen peroxide (9%). Conversely, the application of GABA treatment at this temperature led to an increase in the content of flavonoids (16%), anthocyanin (17%), and the level of total protein by 46% (Fig 1B). When neither SA nor GABA treatments were applied at 27°C, the enzyme activities and pigment levels described above were at moderate levels. However, when SA treatment was applied at 23°C, peroxidase activity (69%) and carotenoid levels (22%) increased. Conversely, when plants were treated with GABA, the enzyme activities and pigment contents decreased (S2 Fig).

### Secondary metabolites correlations with SA and GABA treatments

The analysis of secondary metabolites at various temperatures revealed that control and GABA-treated plants at 27°C exhibited high concentrations of Thymol and p-Cymene. Although the values were slightly higher in control plants compared to GABA-treated plants, the difference was not statistically significant (Fig 1C). However, it was discovered that SA treatment at 23°C increased the levels of Thymol methyl ether (93%) and Carvacrol methyl ether (7%). Moreover, Carvacrol levels were also elevated with SA treatment at 27°C, but the levels of γ-Terpinene were more pronounced in GABA-treated plants at 23°C (Fig 1C).

### Enzymatic characteristics and pigments with SA and GABA treatments in salinity condition

**Fig 2:**
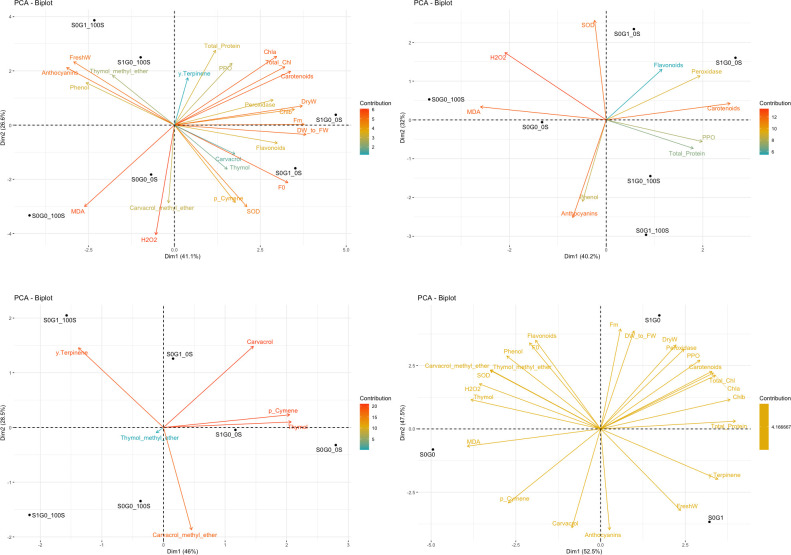
A: Biplot for the average of all measured traits at 0 and 100 mM NaCl. B: Biplot diagram for the average of traits related to the antioxidant status at 0 and 100 mM NaCl. C: Biplot diagram for the average of traits related to the production of secondary metabolites at 0 and 100 mM NaCl. D: Biplot for the average of all measured traits after aggregation of the effects of salt and temperature treatments. S0: no SA, S1: 1 mM SA, G0: no GABA, G1: 1 mM GABA, 0: no NaCl, 100: 100 mM NaCl.

The biplot analysis of the mean of all traits measured at 0 and 100 mM NaCl concentrations from the applied external treatments indicates that the increment of salt concentration resulted in a distinct separation of the studied traits from the control group in all applied external treatments (Fig 2A).

According to the biplot of the mean of enzyme activities and pigment levels at in 0 and 100 mM NaCl concentrations, it was discovered that application of SA treatment in the salt control condition resulted in enhancement of peroxidase activity as well as carotenoids and flavonoids contents (Fig 2B).

Furthermore, the application of 100 mM NaCl concentration increased the activity of polyphenol oxidase and the total protein content in the plant. Similarly, GABA treatment at the same salt concentration triggered a reaction similar to SA treatment, although with less intensity. The absence of both SA or GABA treatments at a concentration of 100 mM salt was associated with higher levels of hydrogen peroxide and malondialdehyde. GABA treatment at 0 mM NaCl enviroment led to an increase in superoxide dismutase activity (Fig 2B).

### Secondary metabolites correlations with SA and GABA treatments in salinity conditions

The study examined the impact of different salt concentrations on the production of secondary metabolites. The results showed that the application of GABA in a 0 mM NaCl environment had the most significant effect on increasing Carvacrol production (Fig 2C).

However, the same treatment at 100 mM NaCl resulted in higher levels of γ-Terpinene production (23%). The highest concentrations of Thymol and p-Cymene were found in the 0 mM NaCl treatment. Although the SA treatment in salt control generated the same reaction in the plant with less intensity. GABA treatment had a greater impact on the increse in Carvacol content compared to SA treatment. Plants that were exposed to 100 mM NaCl and treated with SA exhibited a significant reduction in all tested secondary metabolites. The majority of the diversity observed in flavonoids can be attributed to the second principal component of PCA, which exhibited a significant positive correlation (*P-value 0*.*05*) with the trait closest to SA treatment, namely biomass. Based on these findings, it can be inferred that SA treatment leads to an increase in flavonoid production.

Furthermore, considering the effects of the treatments after combining the impacts of salt and temperature yielded some interesting findings. Based on the observed characteristics, it is apparent that the effects of the treatments can be divided into three groups: control, SA-treated and GABA-treated. The principal component analysis revealed that the first and second components accounted for the entire variation. Low values in the control area, are found in the first and second components. Moreover, highest levels of malondialdehyde, p-Cymene, and Carvacrol could be found in this area. The GABA-treated group was located in the sector that was most explained with the first component. This sector showed a significant increase in the amount of γ -Terpinene and anthocyanins as well as a substantial increase in fresh plant weight.

In contrast, the region explained by the first and second components was the same area where the SA was applied. This treatment resulted in increased peroxidase and polyphenol oxidase activities, carotenoids, chlorophyll content, and plant dry weight. However, there was no change in superoxide dismutase activity, the amount of Carvacrol methyl ether, Thymol methyl ether and total phenol content as due to the above treatments, particularly GABA (Fig 2D).

## Discussion

Seasonal growth and the distribution of plants are regulated by temperature. The greenhouse gas emissions increased the Earth’s average surface temperature by 0.72°C from 1951 to 2012. By the end of this century, the Earth’s average surface temperature could rise by 3.7°C. Increasing temperatures will speed up seed germination, limit vegetative growth, stimulate early blooming, and disturb seasonal growth rhythm. The extinction is already a real possibility for some species that cannot alter the timing of their flowering cycle [7,28–30].

One of the most problematic consequences of global warming is the reduction of agricultural productivity. The 1°C rise will reduced the glycine max yields by 6%. Between 1980 and 2008, global production of maize and wheat decreased by 3.8% and 5.5%, respectively. Sustainable food security depends on having a deep knowledge of the effect of high temperatures on plant growth and development [31,32].

When it comes to plant growth, the intensity, duration, and rate of temperature increase all play a role in the impact of high temperatures. It is impossible to predict how a given plant species or cultivar will be affected by high temprature (HT). Intracellular processes like photosynthesis are susceptible to HT. HT stress reduces photosynthetic pigment levels and affects photosystem II by altering chloroplast shape and inactivating Rubisco. Moreever, HT could alter the transcriptome and metabolome in plants [33–35]. We studied several traits of oregano plant in this study. The results showed that all the studied traits were affected by temperature changes.

The fast rise in temperature (28–37°C for Arabidopsis) causes a heat shock. Arabidopsis could still grow at this level, but there are some issues, particularly with reproductive development, early abortion, anther opening, pollen germination, pollen tube growth inhibition, and stoppage of male and female gametophyte growth. Consequently, fertility plummets resulting in significant yield losses. Seed vigor, germination, root and shoot growth, blooming, and grain filling are all affected. Temperatures above 40°C lead to cell damage and death. Plants develop resistance to lethal HTs after exposure to non-lethal HTs for an extended time. Increased temperatures trigger the development of heat shock proteins and ROS-scavenging enzymes in plants via signal transduction networks [36–38]. Numerous studies have demonstrated the role of ROS and NO regulatory systems in plant heat stress responses. ROS signaling molecules regulate many biological processes, including growth, development, and responses to biotic and abiotic stress [39]. ROS scavenging systems have been demonstrated to have a role in protecting plants against heat stress. Heat shock factors activation demands the production of reactive oxygen species (ROS), which can be produced by heat stress [40]. ROS production and scavenging appear to be important in plant heat responses.

We evaluated the treatments of salicylic acid and gamma aminobutyric acid with both temperature stress and saline environment. Eventually, the results indicated that these plant compounds protect against environmental stresses in *O*. *vulgare*. Notably, SA in temperature stress and GABA in saline environments showed a better protective effect. However, both biomolecules showed significant and acceptable results for protecting the plant. This can be an excellent help to increase the survival and health of the oregano.

SA has been identified as a crucial plant hormone regulating plant immunity since its discovery as an elicitor of tobacco plant resistance to the Tobacco Mosaic Virus (TMV) in 1979. Recent research shows that SA can control abiotic stress tolerance, plant growth and development and interaction with soil microbiome [41,42]. By altering physiological and metabolic processes SA may help plants cope with environmental stresses. Exogenouse SA application changed the yield of essential oils and the activity of antioxidant enzymes in Cornmint and *Mentha × piperita* and alleviated the effects of heat stress [43]. In Rosa hybrida ‘Carolla’ plant SA promoted photosynthesis, antioxidant enzymes activities and osmoregulation followed by quenching the effects of heat stress. In addition, foliar sprays of SA had protective effect on cell membrane structural stability [44]. The same results were reported in *Saponaria officinalis*. SA application caused an additional enhancement in photosynthetic rate, osmoprotectants, and antioxidant levels in salinity stress. It also modulates ion homeostasis and ameliorates salinity tolerance in *Saponaria officinalis* [45].

By interacts with plant growth regulators GABA improves plant stress tolerance. It plays a crucial role in stabilizing the intracellular pH of cells, controlling carbon and nitrogen metabolism, and accumulating osmoprotectant components [16,18,46]. Physiological and proteomic studies reveal GABA priming improved thermotolerance in creeping bentgrass (*Agrostis stolonifera*). *A*. *stolonifera* plants treated with GABA had higher levels of endogenous GABA, photochemical efficiency, performance index on an absorption basis, membrane stability, and osmotic adjustment (OA) after 18 days of heat stress. Treatment by GABA increased the expression of heat shock response proteins (HSP90, HSP70, and HSP16.9) and antioxidant enzymes (SOD and APX) in response to heat stress [47]. GABA pretreatment could induce endogenous GABA levels and subsequently improve physiological effects associated with high temperature in *Trifolium repens* plants. Pretreatment with GABA considerably regulated osmotic adjustment, enhanced photosynthetic, improve cell membrane stability, reduced oxidative damage and increased plant tolerance to HT stress [48]. GABA treatment had healing effects such as improving germination rate, seedling length, fresh and dry weights of the shoot, carbon and nitrogen concentrations in 175 mM NaCl- treated *Lolium perenne* L. [49]. It is reported that the concentration of proline increased in strawberry cv. Aromas by GABA application which could help plants adapt under salinity conditions. GABA application also significantly enhanced the activity of antioxidant enzymes and decreased H_2_O_2_ and MDA content in salt-traeted plants [50]. The study on maize seedling showed that the application of GABA increased seedling growthe rate and chlorophyll content compared to untreated seedlings under the highest saline level. In addition, GABA improved membrane stability and antioxidant enzymes activities under the highest stress level. In maize seedlings that were treated with 1 mM GABA relatively high K^+^ concentration and relatively low Na^+^ concentration were correlated with higher levels of expression of the potassium transporter protein (*ZmHKT1*) gene, and lower expression of the *ZmSOS1* and *ZmNHX1* genes, compared to untreated seedlings [51].

## Conclusion

The present study investigated pigment-enzymatic characteristics and secondary metabolites in *O*. *vulgare*. In general, the results showed that SA in temperature resistance and GABA in stress with saline environment have a better protective effect for developing oregano plants. The use of these two products can be of great help in protecting the plant and improving its propagation in different climatic conditions. However, there is still much work to be done to find the correct connections and signal pathways that oregano uses to protect itself.

## Supporting information

S1 Fig GC-MS chromatogram of *Origanum vulgare* L. (TIF)

S2 Fig The effect of different treatments on some measured traits of *Origanum vulgare* L. S0: no SA, S1: 1 mM SA, G0: no GABA, G1: 1 mM GABA, 0: no NaCl, 100: 100mM NaCl, 23: 23°C, 27: 27°C. Different letters are significantly different according to an Duncan’s test at p < 0.05.
(PDF)

S1 Table Chemical composition of *Origanum vulgare* L. essential oil identified by GC-MS. (PDF)

## References

[1] Kosakowska O, Węglarz Z, Bączek K. Yield and quality of ‘Greek oregano’(*Origanum vulgare* L. subsp. hirtum) herb from organic production system in temperate climate. Ind. Crops. Prod. 2019; 141:111782.

[2] Khan M, Khan ST, Khan M, Mousa AA, Mahmood A, Alkhathlan HZ. Chemical diversity in leaf and stem essential oils of *Origanum vulgare* L. and their effects on microbicidal activities. AMB Express. 2019; 9(1):1–5. doi: 10.1186/s13568-019-0893-3 31673872 PMC6823331

[3] Oniga I, Pușcaș C, Silaghi-Dumitrescu R, Olah NK, Sevastre B, Marica R, et al. *Origanum vulgare* ssp. vulgare: Chemical composition and biological studies. Molecules. 2018; 23(8):2077. 30126246 10.3390/molecules23082077 PMC6222339

[4] de Oliveira AA, França LP, Ramos AD, Ferreira JL, Maria AC, Oliveira KM, et al. Larvicidal, adulticidal and repellent activities against *Aedes aegypti* L. of two commonly used spices, *Origanum vulgare* L. and *Thymus vulgaris L*. S. Afr. J. Bot. 2021; 140:17–24.

[5] Mukhopadhyay R, Sarkar B, Jat HS, Sharma PC, Bolan NS. Soil salinity under climate change: Challenges for sustainable agriculture and food security. J. Environ. Manage. 2021; 280:111736. doi: 10.1016/j.jenvman.2020.111736 33298389

[6] Raza A, Tabassum J, Fakhar AZ, Sharif R, Chen H, et al. Smart reprograming of plants against salinity stress using modern biotechnological tools. Crit. Rev. Biotechnol. 2022; (12):1–28. doi: 10.1080/07388551.2022.2093695 35968922

[7] Raza A, Charagh S, Abbas S, Hassan MU, Saeed F, Haider S, et al. Assessment of proline function in higher plants under extreme temperatures. Plant Biol. 2023; 25(3):379–95. doi: 10.1111/plb.13510 36748909

[8] Mareri L, Parrotta L, Cai G. Environmental Stress and Plants. Int. J. Mol. Sci. 2022; 23(10):5416. doi: 10.3390/ijms23105416 35628224 PMC9141089

[9] Li N, Euring D, Cha JY, Lin Z, Lu M, Huang LJ, et al. Plant hormone-mediated regulation of heat tolerance in response to global climate change. Front. Plant Sci. 2021; 11:627969. doi: 10.3389/fpls.2020.627969 33643337 PMC7905216

[10] Raza A, Charagh S, Najafi-Kakavand S, Abbas S, Shoaib Y, Anwar S, et al. Role of phytohormones in regulating cold stress tolerance: Physiological and molecular approaches for developing cold-smart crop plants. Plant Stress. 2023; (23):100152.

[11] Samad A, Shaukat K, Ansari MU, Nizar M, Zahra et al. Role of foliar spray of plant growth regulators in improving photosynthetic pigments and metabolites in Plantago ovata (Psyllium) under salt stress–A field appraisal. Biocell. 2023;47(3):523–32.

[12] Shaukat K, Baksh G, Zahra N, Hafeez MB, Raza A, Samad A, Nizar M, Wahid A. Foliar application of thiourea, salicylic acid, and kinetin alleviate salinity stress in maize grown under etiolated and de-etiolated conditions. Discover Food. 2022; 26;2(1):27.

[13] Hayat Q, Hayat S, Irfan M, Ahmad A. Effect of exogenous salicylic acid under changing environment: a review. Environ. Exp. Bot. 2010; 68(1):14–25.

[14] Khan MI, Jalil SU, Chopra P, Chhillar H, Ferrante A, Khan NA, etal. Role of GABA in plant growth, development and senescence. Plant Gene. 2021; 26:100283.

[15] Hasan MM, Alabdallah NM, Alharbi BM, Waseem M, Yao G, Liu XD, et al. GABA: A key player in drought stress resistance in plants. Int. J. Mol. Sci. 2021; 22(18):10136. doi: 10.3390/ijms221810136 34576299 PMC8471019

[16] Jalil SU, Ansari MI. Physiological role of Gamma-aminobutyric acid in salt stress tolerance. In: Hasanuzzaman M, Tanveer M, editors. Salt and Drought Stress Tolerance in Plants: Signaling Networks and Adaptive Mechanisms. Springer, Cham. 2020 (pp. 337–350).

[17] Wang Y, Gu W, Meng Y, Xie T, Li L, Li J, etal. γ-Aminobutyric acid imparts partial protection from salt stress injury to maize seedlings by improving photosynthesis and upregulating osmoprotectants and antioxidants. Sci. Rep. 2017; 7(1):1–3. 28272438 10.1038/srep43609 PMC5341084

[18] Raza A, Salehi H, Rahman MA, Zahid Z, Madadkar Haghjou M, Najafi-Kakavand S, et al. Plant hormones and neurotransmitter interactions mediate antioxidant defenses under induced oxidative stress in plants. Front. Plant Sci. 2022; 13:961872. doi: 10.3389/fpls.2022.961872 36176673 PMC9514553

[19] Mahmoodi Tarkhorani S, Sanjarian Dehaghani F, Monsef Shokri M. The effect of salicylic acid treatment on the antioxidant enzyme activities in Thymus vulgaris seedlings. Modares J. Biotech. 2019; 10(1):37–44.

[20] Wellburn AR. The spectral determination of chlorophylls a and b, as well as total carotenoids, using various solvents with spectrophotometers of different resolution. J. Plant Physiol. 1994; 144(3):307–13.

[21] Rahnama H, Ebrahimzadeh H. The effect of NaCl on antioxidant enzyme activities in potato seedlings. Biol. Plant. 2005; 49(1):93–7.

[22] Muthukumarasamy M, Gupta SD, Panneerselvam R. Enhancement of peroxidase, polyphenol oxidase and superoxide dismutase activities by triadimefon in NaCl stressed Raphanus sativus L. Biol. Plant. 2000; 43(2):317–20.

[23] Singleton VL, Rossi JA. Colorimetry of total phenolics with phosphomolybdic-phosphotungstic acid reagents. Am. J. Enol. Vitic. 1965; 16(3):144–58.

[24] Chang CC, Yang MH, Wen HM, Chern JC. Estimation of total flavonoid content in propolis by two complementary colorimetric methods. J. Food Drug Anal. 2002; 10(3).

[25] Jahantigh O, Najafi F, Badi HN, Khavari-Nejad RA, Sanjarian F. Changes in antioxidant enzymes activities and proline, total phenol and anthocyanine contents in Hyssopus officinalis L. plants under salt stress. Acta Biol. Hung. 2016; 67(2):195–204. doi: 10.1556/018.67.2016.2.7 27165530

[26] Velikova V, Yordanov I, Edreva A. Oxidative stress and some antioxidant systems in acid rain-treated bean plants: protective role of exogenous polyamines. Plant Sci. 2000; 151(1):59–66.

[27] Kassambara A. Practical guide to cluster analysis in R: Unsupervised machine learning. Vol. 1. Sthda; 2017.

[28] Willis CG, Ruhfel B, Primack RB, Miller-Rushing AJ, Davis CC. Phylogenetic patterns of species loss in Thoreau’s woods are driven by climate change. PNAS. 2008; 105(44):17029–33. doi: 10.1073/pnas.0806446105 18955707 PMC2573948

[29] Raza A, Razzaq A, Mehmood SS, Zou X, Zhang X, Lv Y, et al. Impact of climate change on crops adaptation and strategies to tackle its outcome: A review. Plants. 2019; 8(2):34. doi: 10.3390/plants8020034 30704089 PMC6409995

[30] Cassia R, Nocioni M, Correa-Aragunde N, Lamattina L. Climate change and the impact of greenhouse gasses: CO2 and NO, friends and foes of plant oxidative stress. Front. Plant Sci. 2018; 9:273. doi: 10.3389/fpls.2018.00273 29545820 PMC5837998

[31] Zhao C, Liu B, Piao S, Wang X, Lobell DB, Huang Y, et al. Temperature increase reduces global yields of major crops in four independent estimates. PNAS. 2017; 114(35):9326–31. doi: 10.1073/pnas.1701762114 28811375 PMC5584412

[32] Shah K, Chaturvedi V, Gupta S. Climate change and abiotic stress-induced oxidative burst in rice. In: Hasanuzzaman M, Fujita M, Nahar K, Biswas JK, editors. Advances in rice research for abiotic stress tolerance. Woodhead Publishing. 2019. pp. 505–535.

[33] Sharkey TD, Zhang R. High temperature effects on electron and proton circuits of photosynthesis. J. Integr. Plant Biol. 2010;52(8):712–22. doi: 10.1111/j.1744-7909.2010.00975.x 20666927

[34] Hasanuzzaman M, Nahar K, Alam MM, Roychowdhury R, Fujita M. Physiological, biochemical, and molecular mechanisms of heat stress tolerance in plants. Int. J. Mol. Sci. 2013; 14(5):9643–84. doi: 10.3390/ijms14059643 23644891 PMC3676804

[35] Raza A, Tabassum J, Kudapa H, Varshney RK. Can omics deliver temperature resilient ready-to-grow crops? Crit. Rev. Biotechnol. 2021;41(8):1209–1232. doi: 10.1080/07388551.2021.1898332 33827346

[36] Sage TL, Bagha S, Lundsgaard-Nielsen V, Branch HA, Sultmanis S, Sage RF. The effect of high temperature stress on male and female reproduction in plants. Field Crops Research. 2015; 182:30–42.

[37] Bahuguna RN, Jagadish KS. Temperature regulation of plant phenological development. Environ. Exp. Bot. 2015; 111:83–90.

[38] Li B, Gao K, Ren H, Tang W. Molecular mechanisms governing plant responses to high temperatures. J. Integr. Plant Biol. 2018; 60(9):757–79. doi: 10.1111/jipb.12701 30030890

[39] Mittler R, Vanderauwera S, Suzuki N, Miller GA, Tognetti VB, Vandepoele K, et al. ROS signaling: the new wave?. Trends Plant Sci. 2011; 16(6):300–9. doi: 10.1016/j.tplants.2011.03.007 21482172

[40] Driedonks N, Xu J, Peters JL, Park S, Rieu I. Multi-level interactions between heat shock factors, heat shock proteins, and the redox system regulate acclimation to heat. Front. Plant Sci. 2015; 6:999. doi: 10.3389/fpls.2015.00999 26635827 PMC4647109

[41] Zhang Y, Li X. Salicylic acid: biosynthesis, perception, and contributions to plant immunity. Curr.Opin. Plant Biol. 2019; 50:29–36. doi: 10.1016/j.pbi.2019.02.004 30901692

[42] Koo YM, Heo AY, Choi HW. Salicylic acid as a safe plant protector and growth regulator. Plant Pathol. J. 2020; 36(1):1. doi: 10.5423/PPJ.RW.12.2019.0295 32089657 PMC7012573

[43] Haydari M, Maresca V, Rigano D, Taleei A, Shahnejat-Bushehri AA, Hadian J, et al. Salicylic acid and melatonin alleviate the effects of heat stress on essential oil composition and antioxidant enzyme activity in Mentha× piperita and Mentha arvensis L. Antioxidants. 2019; 8(11):547. 31766277 10.3390/antiox8110547 PMC6912601

[44] Wang K, Shen Y, Wang H, He S, Kim WS, Shang W, et al. Effects of Exogenous Salicylic Acid (SA), 6-Benzylaminopurine (6-BA), or Abscisic Acid (ABA) on the Physiology of Rosa hybrida ‘Carolla’under High-Temperature Stress. Horticulturae. 2022; 8(9):851.

[45] Xu L, Chen H, Zhang T, Deng Y, Yan J, Wang L. Salicylic Acid improves the salt tolerance capacity of Saponaria officinalis by modulating its photosynthetic rate, osmoprotectants, antioxidant levels, and ion homeostasis. Agronomy. 2022; 12(6):1443.

[46] Hijaz F, Nehela Y, Killiny N. Application of gamma-aminobutyric acid increased the level of phytohormones in Citrus sinensis. Planta. 2018; 248(4):909–18. doi: 10.1007/s00425-018-2947-1 29961199

[47] Li Z, Zeng W, Cheng B, Huang T, Peng Y, Zhang X. γ-Aminobutyric acid enhances heat tolerance associated with the change of proteomic profiling in creeping bentgrass. Molecules. 2020; 25(18):4270. 32961841 10.3390/molecules25184270 PMC7571209

[48] Qi H, Kang D, Zeng W, Jawad Hassan M, Peng Y, Zhang X, et al. Alterations of endogenous hormones, antioxidant metabolism, and aquaporin gene expression in relation to γ-aminobutyric acid-regulated thermotolerance in white clover. Antioxidants. 2021; 10(7):1099. 34356332 10.3390/antiox10071099 PMC8301151

[49] Tang J, Li M, Mao P, Jiang Y. Effects of Gamma-Aminobutyric Acid on Seed Germination, Ion Balance, and Metabolic Activity in Perennial Ryegrass Under Salinity Stress. J Plant Growth Regul. 2022; 41(4):1835–44.

[50] Golnari S, Vafaee Y, Nazari F, Ghaderi N. Gamma-aminobutyric acid (GABA) and salinity impacts antioxidative response and expression of stress-related genes in strawberry cv. Aromas. Braz. J. Bot. 2021; 44(3):639–51.

[51] Aljuaid BS, Ashour H. Exogenous γ-Aminobutyric Acid (GABA) Application Mitigates Salinity Stress in Maize Plants. Life. 2022; 12(11):1860. 36430995 10.3390/life12111860 PMC9697566

