# Supplementary figures and images for: The role of γ-aminobutyric acid and salicylic acid in heat stress tolerance under salinity conditions in Origanum vulgare L
Source: PLoS One. 2023 Jul 7;18(7):e0288169. doi: 10.1371/journal.pone.0288169 (PMC10328350; doi:10.1371/journal.pone.0288169)

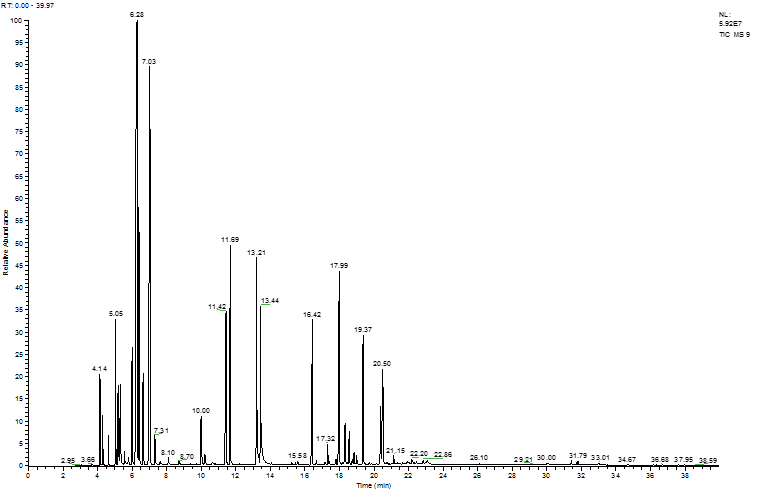

Supplement: S1 Fig — (TIF) [file pone.0288169.s001.tif]
