# Supplementary material for: The role of γ-aminobutyric acid and salicylic acid in heat stress tolerance under salinity conditions in Origanum vulgare L
Source: PLoS One. 2023 Jul 7;18(7):e0288169. doi: 10.1371/journal.pone.0288169 (PMC10328350; doi:10.1371/journal.pone.0288169)
Supplement: S2 Fig — S0: no SA, S1: 1 mM SA, G0: no GABA, G1: 1 mM GABA, 0: no NaCl, 100: 100mM NaCl, 23: 23°C, 27: 27°C. Different letters are significantly different according to an Duncan’s test at p < 0.05. (PDF) [file pone.0288169.s002.pdf]

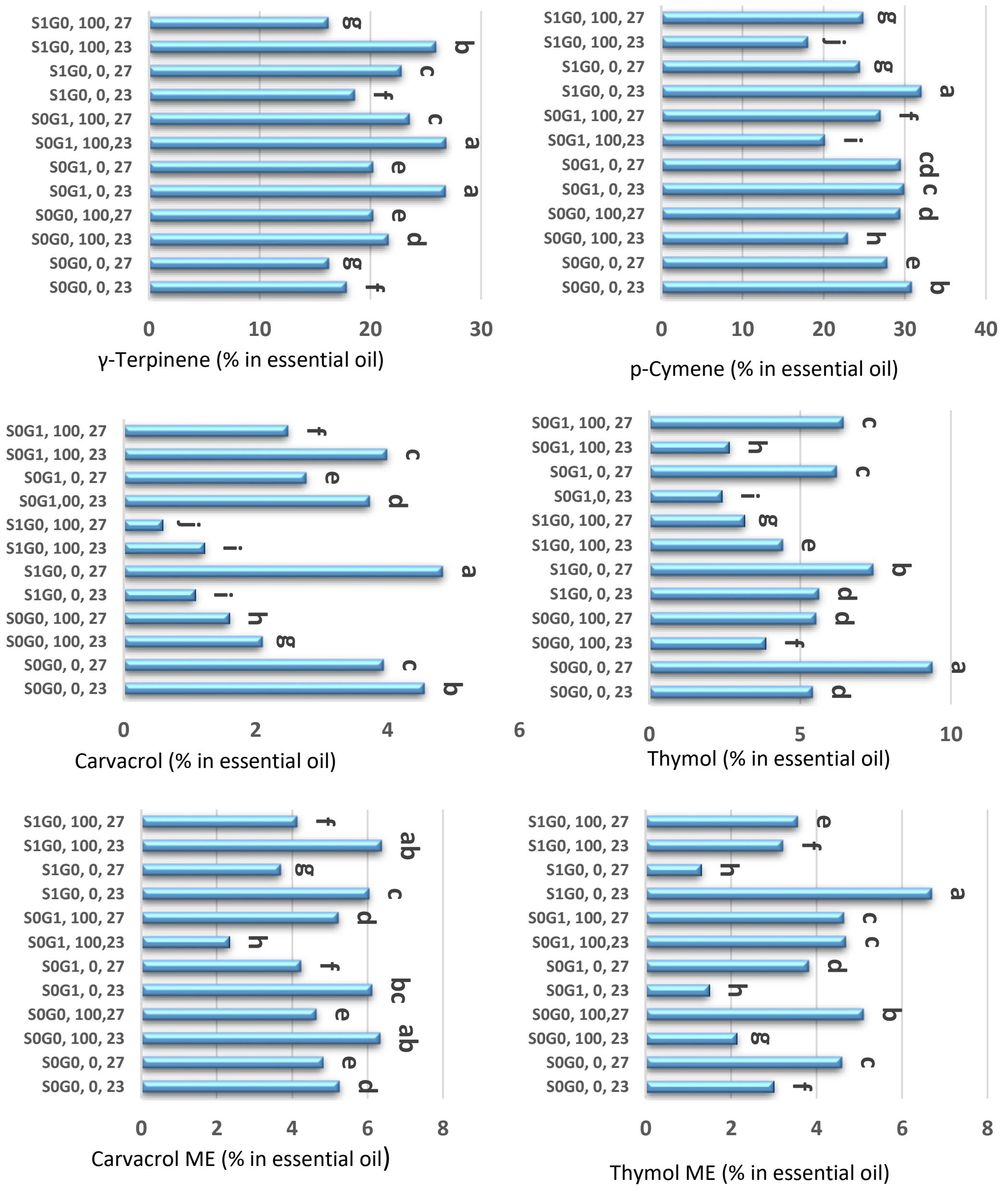

S1G0, 100, 27
S1G0, 100, 23
S1G0, 0, 27
S1G0, 0, 23
S0G1, 100, 27
S0G1, 100,23
S0G1, 0, 27
S0G1, 0, 23
S0G0, 100,27
S0G0, 100, 23
S0G0, 0, 27
S0G0, 0, 23
0 10 20 30
γ-Terpinene (% in essential oil)
S1G0, 100, 27
S1G0, 100, 23
S1G0, 0, 27
S1G0, 0, 23
S0G1, 100, 27
S0G1, 100,23
S0G1, 0, 27
S0G1, 0, 23
S0G0, 100,27
S0G0, 100, 23
S0G0, 0, 27
S0G0, 0, 23
0 10 20 30 40
p-Cymene (% in essential oil)
S0G1, 100, 27
S0G1, 100, 23
S0G1, 0, 27
S0G1,00, 23
S1G0, 100, 27
S1G0, 100, 23
S1G0, 0, 27
S1G0, 0, 23
S0G0, 100, 27
S0G0, 100, 23
S0G0, 0, 27
S0G0, 0, 23
0 2 4 6
Carvacrol (% in essential oil)
S0G1, 100, 27
S0G1, 100, 23
S0G1, 0, 27
S0G1,0, 23
S1G0, 100, 27
S1G0, 100, 23
S1G0, 0, 27
S1G0, 0, 23
S0G0, 100, 27
S0G0, 100, 23
S0G0, 0, 27
S0G0, 0, 23
0 5 10
Thymol (% in essential oil)
S1G0, 100, 27
S1G0, 100, 23
S1G0, 0, 27
S1G0, 0, 23
S0G1, 100, 27
S0G1, 100,23
S0G1, 0, 27
S0G1, 0, 23
S0G0, 100,27
S0G0, 100, 23
S0G0, 0, 27
S0G0, 0, 23
0 2 4 6 8
Carvacrol ME (% in essential oil)
S1G0, 100, 27
S1G0, 100, 23
S1G0, 0, 27
S1G0, 0, 23
S0G1, 100, 27
S0G1, 100,23
S0G1, 0, 27
S0G1, 0, 23
S0G0, 100,27
S0G0, 100, 23
S0G0, 0, 27
S0G0, 0, 23
0 2 4 6 8
Thymol ME (% in essential oil)

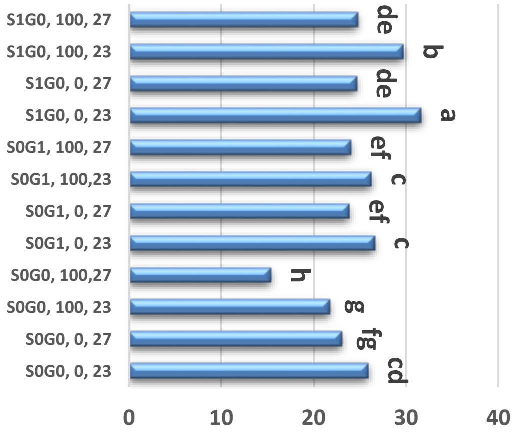

chlorophyll a µg/mg Fw

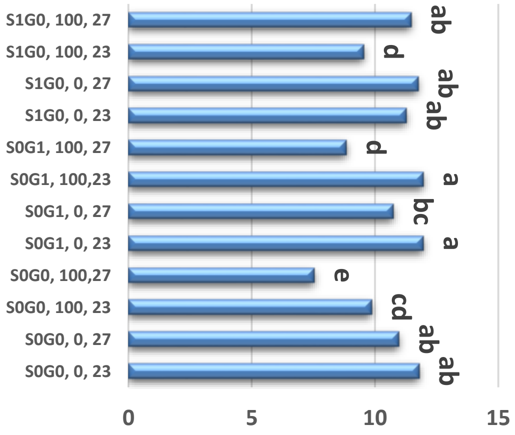

chlorophyll b µg/mg Fw

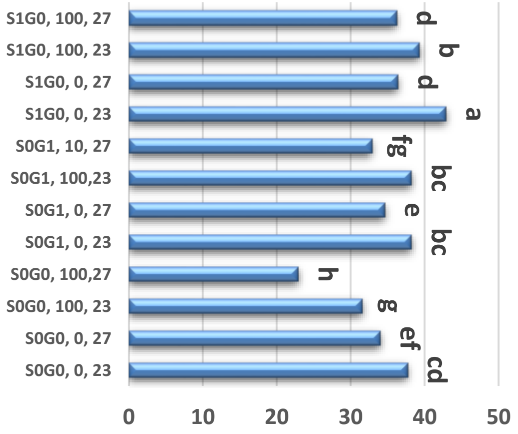

Total chlorophyll µg/mg Fw

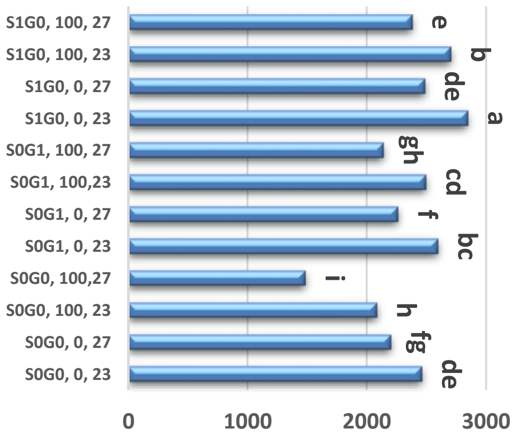

Total carotenoids µg/mg Fw

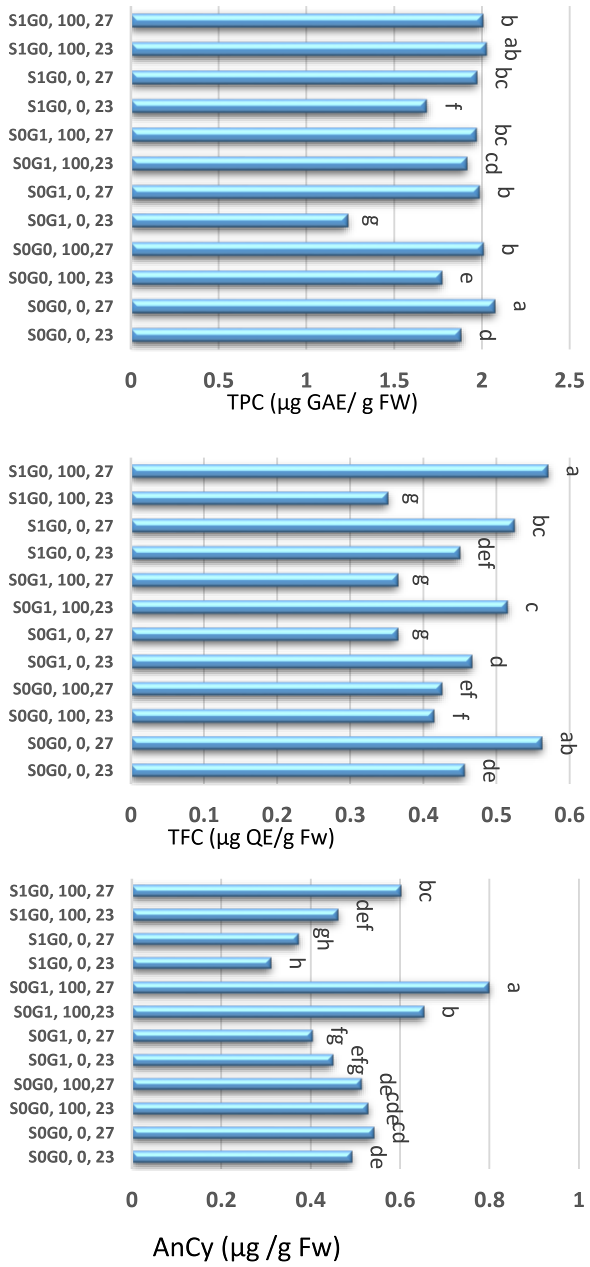
S1G0, 100, 27
S1G0, 100, 23
S1G0, 0, 27
S1G0, 0, 23
S0G1, 100, 27
S0G1, 100,23
S0G1, 0, 27
S0G1, 0, 23
S0G0, 100,27
S0G0, 100, 23
S0G0, 0, 27
S0G0, 0, 23
0 0.5 1 1.5 2 2.5
TPC (µg GAE/ g FW)
0 0.1 0.2 0.3 0.4 0.5 0.6
TFC (µg QE/g Fw)
0 0.2 0.4 0.6 0.8 1
AnCy (µg /g Fw)

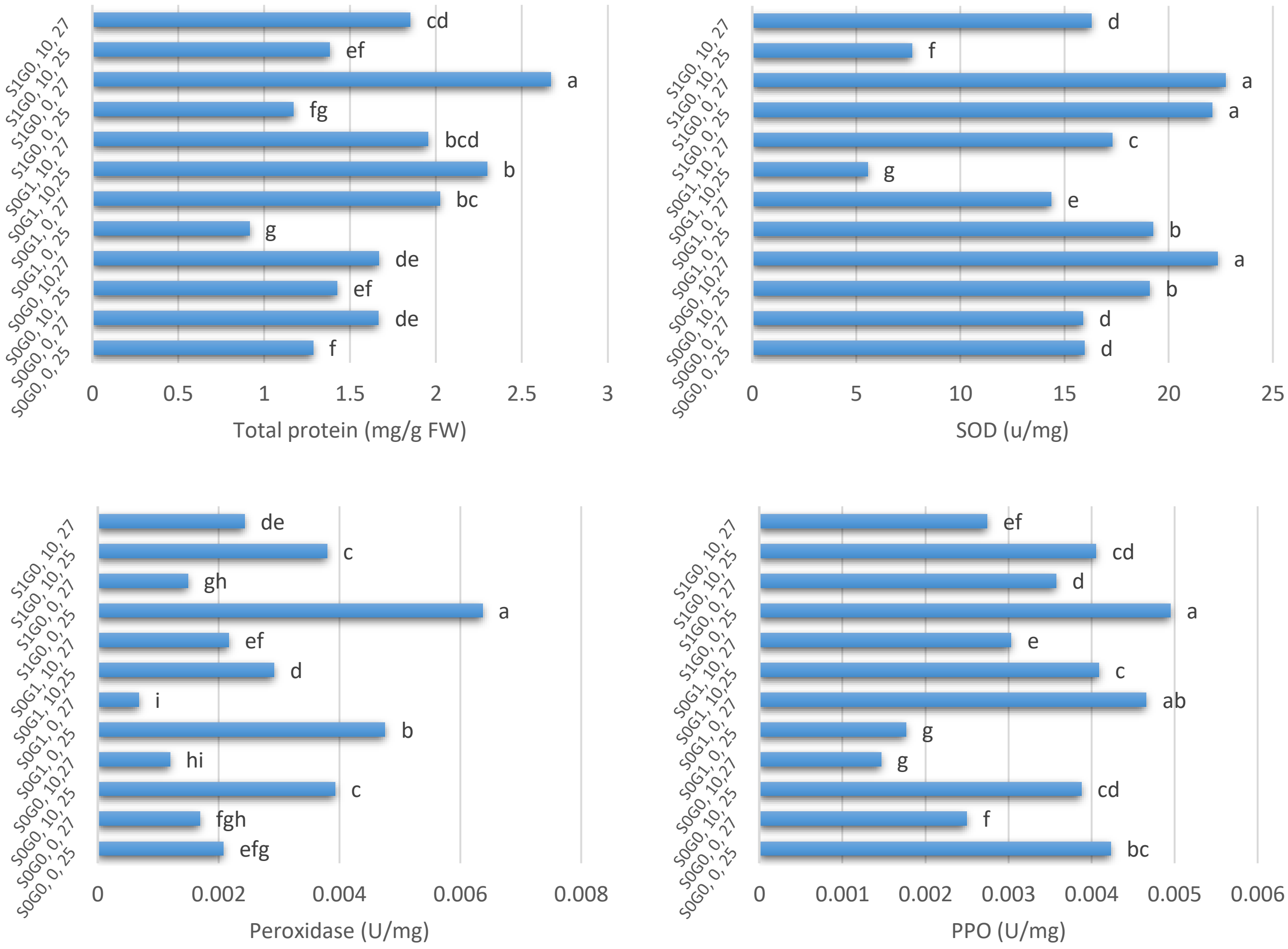

S2 Fig. The effect of different treatments on some measured traits of *Origanum vulgare* L. S0: no SA, S1: 1 mM SA, G0: no GABA, G1: 1 mM GABA, 0: no NaCl, 100: 100mM NaCl

Different letters are significantly different according to an Duncan's test at $p < 0.05$
