# Supplementary material for: The role of γ-aminobutyric acid and salicylic acid in heat stress tolerance under salinity conditions in Origanum vulgare L
Source: PLoS One. 2023 Jul 7;18(7):e0288169. doi: 10.1371/journal.pone.0288169 (PMC10328350; doi:10.1371/journal.pone.0288169)
Supplement: S1 Table — (PDF) [file pone.0288169.s003.pdf]

Table S1 - Chemical composition of *Origanum vulgare* L. essential oil identified by GC-MS

N°	Compound	GC%	RT
1	α-thujene	1.3019	5.71
2	α-Pinene	0.7375	5.91
3	Camphene	0.5045	6.33
4	Sabinene	2.6388	6.81
5	1-Octen-3-ol	1.3961	7.08
6	β-Myrcene	3.1166	7.22
7	3-Octanol	0.3081	7.61
8	α-Terpinene	2.2297	7.87
9	p-Cymene	30.8086	8.25
10	Ocimene <(Z)-β->	3.5067	8.28
11	Ocimene <(E)-β->	1.7894	8.53
12	γ-Terpinene	17.834	9.01
13	Sabinene hydrate <cis->	0.5776	9.6
14	Borneol	0.847	12.59
15	Thymol, methyl ether	2.9959	13.67
16	Carvacrol, methyl ether	5.2574	13.93
17	Thymol	5.4178	16.12
18	Carvacrol	4.5649	16.37
19	(E)-caryophyllene	2.9987	18.52
20	α-Humulene	0.2771	19.43
21	Germacrene D	3.2935	20.08
22	Bicyclogermacrene	0.6406	20.43
23	Bisabolene <β->	0.6855	20.49
24	Cadinene <δ->	0.6433	20.85
25	Sesquiphellandrene <β->	0.2667	20.93
26	Bisabolene <(E)-α->	2.3378	21.27
27	Spathulenol	3.0243	22.76

GC-MS: gas chromatography-mass spectrometry; RT: retention time
